# Antitumor Activity of Protons and Molecular Hydrogen: Underlying Mechanisms

**DOI:** 10.3390/cancers13040893

**Published:** 2021-02-20

**Authors:** Luc Rochette, Marianne Zeller, Yves Cottin, Catherine Vergely

**Affiliations:** 1 PEC2 (EA7460), Faculty of Health Sciences, University Bourgogne-Franche Comte, 7 Bd Jeanne d’Arc, 21000 Dijon, France; marianne.zeller@u-bourgogne.fr (M.Z.); yves.cottin@chu-dijon.fr (Y.C.); cvergely@u-bourgogne.fr (C.V.); 2 Department of Cardiology, University Hospital of Dijon, 21000 Dijon, France

**Keywords:** molecular hydrogen, antioxidant, cancer

## Abstract

**Simple Summary:**

Protons (H^+^) and molecular hydrogen (H_2_) in the cell are critical in a wide variety of processes. New cancer treatment uses H_2_, a biologically inactive gas. H_2_ can rapidly penetrate cell membranes and reach subcellular components to protect nuclear DNA and mitochondria. H_2_ reduces oxidative stress, exerts anti-inflammatory effects, and acts as a modulator of apoptosis. Exogenous H_2_ is a protective therapy that can be used in cancer. Cyclotrons and synchrotrons are currently used to produce protons. Proton beam radiotherapy (PBT) offers great promise for the treatment of a wide variety of cancers. H_2_ and different types of H_2_ donors may represent a novel therapeutic strategy in cancer treatment.

**Abstract:**

Understanding the structure and dynamics of the various hydrogen forms has been a subject of numerous studies. Protons (H^+^) and molecular hydrogen (H_2_) in the cell are critical in a wide variety of processes. A new cancer treatment uses H_2_, a biologically inactive gas. Due to its small molecular weight, H_2_ can rapidly penetrate cell membranes and reach subcellular components to protect nuclear DNA and mitochondria. H_2_ reduces oxidative stress, exerts anti-inflammatory effects, and acts as a modulator of apoptosis. Exogenous H_2_, administered by inhalation, drinking H_2_-rich water, or injecting H_2_-rich saline solution, is a protective therapy that can be used in multiple diseases, including cancer. In particle therapy, cyclotrons and synchrotrons are the accelerators currently used to produce protons. Proton beam radiotherapy (PBT) offers great promise for the treatment of a wide variety of cancers due to the sharp decrease in the dose of radiation at a defined point. In these conditions, H_2_ and different types of H_2_ donors may represent a novel therapeutic strategy in cancer treatment.

## 1. Introduction

Oxidation and reduction are ubiquitous reactions that play key roles in the chemistry of aerobic organisms. Disturbances in the cellular redox balance have been related to pro-aging mechanisms and an increased risk of diseases such as cancers. A shift in the balance between oxidants and antioxidants in favor of oxidants is known as “oxidative stress”. Oxidative stress (OS) is a deviation from the steady redox state, and it indicates an imbalance due to excess levels of reactive oxygen species (ROS) or oxidants that surpass the cell’s capability to provide an efficient antioxidant response [1]. The dysregulation of cellular pH is a well-known characteristic of malignancy. Both hydrogen transport and cytoplasmic pH play critical roles in the management of cell growth and proliferation, and tumorigenesis. In the cell, protons (H^+^) and molecular hydrogen (H_2_) are critical in a wide variety of processes. Recently, H_2_ has been studied in preclinical and clinical trials on various diseases associated with oxidative and inflammatory stress. In this context, there has been much interest in H_2_ and H^+^ as possible therapeutic approaches in cancer. This review describes the role of H_2_ and H^+^ in relation to OS and outlines the potential anticancer activity of this endogenous ion and different types of H_2_ donors.

## 2. Background: The Different Forms of Oxygen and Hydrogen

The structure and dynamics of the different forms of hydrogen and oxygen are the subjects of numerous studies. When oxygen combines with another element, referred to as oxidation, more energy is released than when any other elements are combined. In the cell, the energy released is slow.

The atomic number of oxygen is 8 on the Periodic Table of Elements, implying that an oxygen atom holds eight electrons. Its electrons are filled in the following order: two electrons in the first orbital, and six electrons in the second orbital. Therefore, there are 16 electrons in the oxygen molecule. Oxygen has two unpaired electrons in separate orbitals in its outer shell. This electronic structure makes oxygen especially susceptible to radical formation. A free radical is defined as any chemical species that contains unpaired electrons in its outer orbital.

Atomic hydrogen (H) is number 1 on the Periodic Table of Elements. It consists of one proton and one unpaired electron, and it is consequently a free radical. When the hydrogen atom loses an electron, all that remains is a proton. It becomes the positively charged hydrogen ion known as H^+^. H_2_ is a gas that forms when two hydrogen atoms bond together and become a hydrogen molecule consisting of two protons and two electrons.

**Figure 1:**
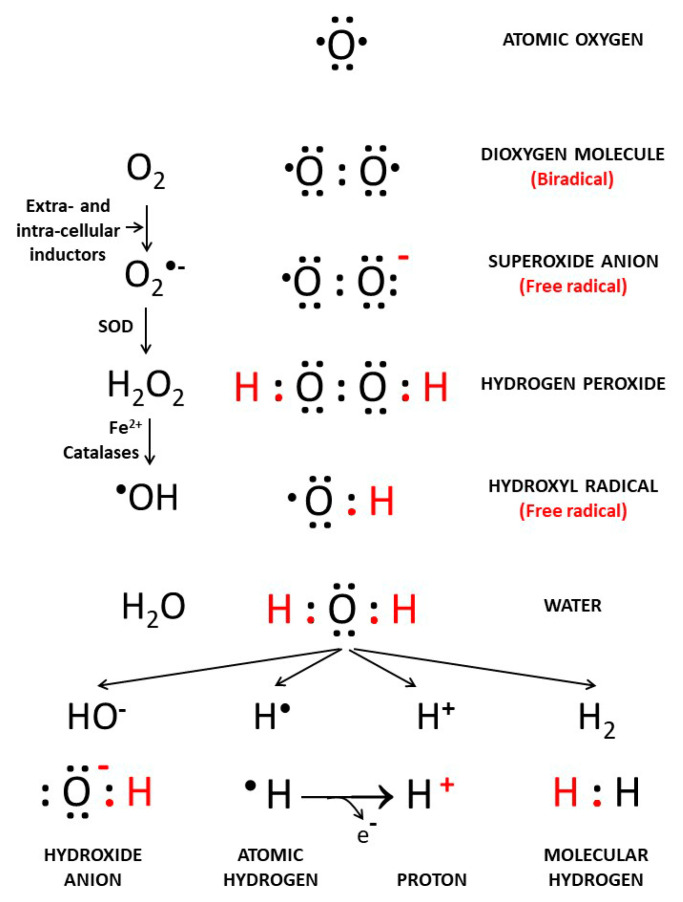
Reduction of O_2_ to H_2_O, free radicals’ intermediates and hydrogen forms. Extra- and intra-cellular inductions interact with atomic oxygen (O_2_) to promote formation of free radical derivatives such as superoxide (O_2_^−•^), hydrogen peroxide (H_2_O_2_), and the highly reactive hydroxyl radical (•OH).

Hydroxide (OH^−^), also known as the hydroxyl ion, is not a free radical. Sequential reduction of molecular oxygen leads to formation of a group of ROS, such as the superoxide anion and hydroxyl radical. Hydroxyl radical •OH is the neutral form of the hydroxide ion (OH^−^) and is a highly reactive free radical. Superoxide anion (O_2_^−•^) is of major importance in cellular biology because it leads to the formation of ROS. O_2_^−•^ can be dismutated to molecular oxygen (O_2_) and hydrogen peroxide (H_2_O_2_), either spontaneously or in a reaction catalyzed by superoxide dismutases (SODs). H_2_O_2_ is converted to •OH via several routes, catalyzed by Haber-Weiss reactions and metal-catalyzed by Fenton reactions (Figure 1). Concerning ROS in biology, a process of chemical chain reaction has been described involving three stages: initiation, propagation, and termination [2].

Protons (H^+^) and hydroxide (OH^−^) ions in the cell are critical for a wide variety of biological processes. Proton transport across the plasma membrane is central for the maintenance of pH. Cells maintain intracellular pH (pHi) within a narrow range (7.1–7.2) by controlling membrane proton pumps and transporters [3]. The normal physiological pH of mammalian arterial blood is maintained at 7.40 ± 0.05, depending in part on several pH buffering systems such as albumin. Body fluid acidosis is involved in the pathogenesis of metabolic diseases. For instance, chronic ketoacidosis is found in diabetes mellitus patients due to increased levels of ketone bodies in the blood [4,5]. In humans, the maintenance of pH in the diverse cell compartments (intracellular and extracellular) is achieved through various regulatory systems. Ions utilize several paths to enter the cytosolic environment. In this area, the bicarbonate ion (HCO_3_^−^) has a fundamental role, and acid–base homeostasis with HCO_3_^−^ is critically regulated in various systems through various transporters, which have been extensively reviewed [6,7]. The acid–base balance is a critical factor in the heart, and, consequently, acid–base imbalance contributes to organ disease [8]. Additionally, it is suggested that pHi plays an essential role in cancer metabolism, where a reverse pH gradient is a hallmark that is evidenced by extracellular acidosis and intracellular alkalization [9].

## 3. Regulation of pH in Cancer Cells

Cancer cells within a tumor are heterogeneous concerning their specificities, such as morphology, cell surface markers, proliferation kinetics, and response to therapy.

As stated above, cells maintain pHi within a narrow range (7.1–7.2), and the physiological pH of arterial blood is maintained at precisely 7.40. In contrast, cancer cells function in alkaline cytoplasmic pH conditions greater than 7.4 and extracellular pH of 6.7–7.1. Thus, it is important to understand how the redox state and pH change during cancer progression, how it activates different proteins during proliferation, and what the mechanisms implicated in the resistant properties of cancers are. There is strong evidence that cancer cells are usually under higher OS than normal cells and that an additional increase in pro-oxidant levels can trigger cell death. A redox paradox has been described in cancer cells, and variations in pH appear to be involved in this process [10].

The acidic extracellular microenvironment in tumor supports the expression of angiogenic factors. Several recent reviews have discussed the oncogenic consequences of the transport of H^+^ in the plasma membrane, and, in this field, there have been numerous fundamental studies underlining the role of endosomal pH in cancer phenotypes. Ion transporters and channels are implicated in the adjustment of endosomal pH throughout the endosomal compartments. Intracellular members of (Na^+^/H^+^) exchangers (NHEs) are a superfamily implicated in tumor metastases [11,12]. These exosomes are important intercellular communication mediators within the organs [13], and they are secreted abundantly by cancer cells. Elevated intracellular Ca^2+^ and modifications of the transport of H^+^ are critical for tumor progression and metastasis. The acid–base balance is controlled with a different approach in cancer cells and in normal cells [14]. Cancer cells have an unusual regulation of proton dynamics associated with a regional hypoxia and increased glycolysis inducing extracellular acidity and intracellular alkalinity. Accumulation of lactate and proton ions in the extracellular space results in an acidic environment that promotes proliferation [15].

The oncogenic transformation modifies the metabolic profile of cells towards an upregulation of glycolysis [16]. The ubiquitous Na^+^/H^+^ exchanger was described as playing a key role in both tumorigenesis and in cell death processes. Altered H^+^ dynamics might be a universal mechanism in environmental carcinogenesis, in which case, the targeting of proton transporters would be a new class of potential anticancer treatments [16].

## 4. Biochemistry of Molecular Hydrogen

H_2_ diffuses into the cytosol and rapidly reaches the nucleus and mitochondria. Endogenous H_2_ is produced by enteral bacteria as a byproduct of anaerobic metabolism in connection with the fermentation of carbohydrates by the resident enterobacterial flora [17]. H_2_ is produced in the gut and is the main gas marker of carbohydrate fermentation. While it is clear that it is produced in the gut, its presence in exhaled breath is still the subject of debate. A breath test consists of administering a carbohydrate (lactulose or glucose) and measuring the exhaled H_2_ gas concentrations over a period of time [18]. Another pathway for bacterial H_2_ production is the cleavage of pyruvate into formate in the human gut, but this pathway has not yet been demonstrated [19]. Hydrogenases either utilize H_2_ as a substrate or produce H_2_ by the reduction of protons through the reaction: 2H^+^ + 2e^−^ ⇌ H_2_.

The microorganisms produce H_2_ via the water–gas shift reaction with carbon monoxide (CO): CO + H_2_O → CO_2_ + H_2_. Some photosynthetic bacteria are unique in that they contain a carbon monoxide (CO) oxidation pathway that converts CO and H_2_O into H_2_ and CO_2_. During the biologically mediated reaction, a carbon monoxide dehydrogenase (CODH) oxidizes CO, and electrons are released. Subsequently, coupled hydrogenase reduces the released electrons to H_2_ [20]. CO is endogenously produced by vascular smooth muscle cells (SMCs) under conditions of hypoxia, and it can then modulate cGMP levels in both endothelial cells and SMCs [5,21].

## 5. Antioxidant and Anti-Inflammatory Properties of H_2_

**Figure 2:**
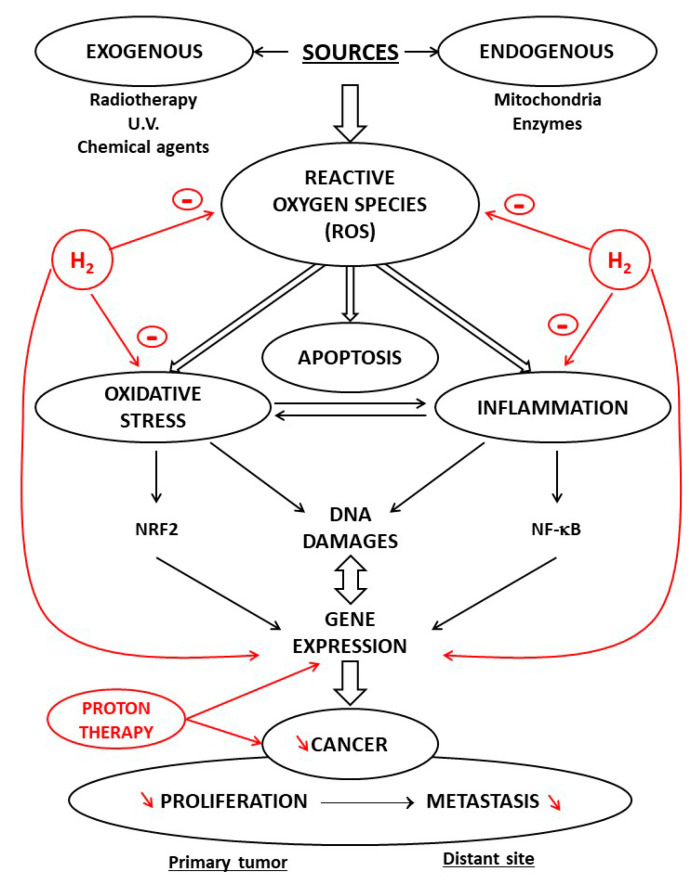
Effects of proton and H_2_ therapies on oxidative stress and inflammation in carcinogenesis. reactive oxygen species (ROS) via oxidative stress and inflammation promote carcinogenesis in cells with defective signaling factors. A crosstalk exists between oxidative stress and inflammation; this interconnection is associated with activation of NRF2 and NF-κB. Protons induce perturbations of gene expression inhibiting the cancer progression processes of invasion. H_2_ reduces oxidative stress, exerts anti-inflammatory effects, and acts as a modulator of apoptosis. It scavenges ROS and, penetrating biomembranes, reaches cell nuclei. It modulates signal transduction implicated in proliferation and metastasis processes.

There are three methods used for hydrogen administration: inhaling hydrogen gas, drinking hydrogen-rich water, or injecting hydrogen-rich saline (Figure 2).

Most studies are based on animal models, which provide a basis for the clinical application of H_2_. The rationale for H_2_ use in clinical medicine is linked to its antioxidant and anti-inflammatory properties [22]. Depending on their reactivity and localization, ROS are involved in physiological and pathophysiological processes, and OS is involved in the initiation and development of inflammation [4]. Numerous reviews in the literature have documented the interconnection between OS and inflammation and the importance of targeting NRF2/antioxidant signaling and NF-κB inflammatory response. H_2_ is now believed to impact these parameters [23].

During tumor metabolic reprogramming, ROS are generated and the antioxidant systems are activated. High levels of ROS lead to oxidative damage and even cell death, whereas ROS at low levels act as second messenger to regulate many signaling pathways in relationship with the development of the cells, resulting in protein conformational and functional alterations. Elevated ROS levels have been found in most cancer cell lines and have been implicated in disease progression and resistance to treatment [24]. An increased level of ROS is common to almost all tumor cells, and this has been suggested as a possible common target for therapeutic approaches [25]. In recent years, new agents with specific antioxidant functions have drawn much attention as potential therapies because of their ability to reduce ROS formation and cancer development [26]. Among these protective agents, H_2_ has unique properties. It contributes to the antioxidant defense in the cell since it scavenges the hydroxyl radical, which is the most cytotoxic ROS. Using the method of spin-trapping by 5,5-dimethyl-1-pyrroline N-oxide (DMPO), it has been demonstrated that H_2_ reacts directly with hydroxyl radicals in a cell-free system and reduces it in cell cultures [27]. H_2_ decreased NADPH oxidase (NOX)-derived free radicals produced during phagocytosis, NADPH oxidase being an important pro-oxidative enzyme [28]. H_2_ decreases OS both directly and also by inducing antioxidant enzymes, such as superoxide oxidase (SOD) and myeloperoxidase [29].

Through which mechanisms is H_2_ an antioxidant? Antioxidant molecules may directly react with reactive radicals and destroy them. Many criteria such as an action on transition metal chelation must be considered when evaluating the antioxidant potential of a compound [30]. Specific antioxidant mechanisms limit oxidative damage, but, in certain conditions, oxidants can have pro-oxidative effects. Some agents act as antioxidants and are able to scavenge free radicals due to their hydrogen-donating ability [31]. The processes underlying the various effects of H_2_ have been investigated, and it has been proven that H_2_, although it depletes ROS, may interfere with defense mechanisms and the activity of the immune system. The observed activities of H_2_ were dose-dependent, and in some cases, they appeared inconsistent or even paradoxical [32].

## 6. Hydrogen Paradox: Antioxidant and/or Prooxidant

The apparent paradox between the protective and toxic effects of H_2_ is supported by experimental and clinical evidence. This paradox has been described for a number of antioxidants compounds. Melatonin is a well-documented antioxidant; however, melatonin enhances ROS formation in a redox system containing low concentrations of copper due to the reduction of cupric to cuprous ion by melatonin [33]. This paradox has also been demonstrated with vitamins such ascorbic acid (vitamin C). Ascorbic acid has numerous antioxidant properties, but it can exert pro-oxidant effects in vitro, usually by interacting with transition metal ions [34]. Transition metal ions are key elements for various biological processes, and their homeostasis is maintained within strict limits through tightly regulated mechanisms redox- and non-redox-metal-induced formation of free radicals and their role in human disease [35].

The relationships between H_2_ transporters and transition metal ions are complex. Biological studies propose that the metal ions affect enzymes such as transhydrogenase by binding to a site in the proton transfer pathway. The inhibition of proton-transfer steps by metal ions such as Zn^2+^ has been described in studies on a number of membrane proteins, such as NADH dehydrogenase and cytochrome oxidase [36,37].

## 7. Proton Radiotherapy and Antitumor Activity

New treatments that rely on hydrogen metabolism appear to offer selective inhibition of cancer cells. For instance, there is evidence that new radiotherapy procedures offer selective inhibition of cancer cells. In particle therapy, cyclotrons and synchrotrons are the currently used accelerators. The major component of a typical cyclotron for therapy is a proton source in the center of the cyclotron, in which hydrogen gas is ionized and from which the protons are extracted. Protons are heavy, positively charged particles that can be stripped from hydrogen gas and accelerated toward the speed of light using an accelerator [22]. Proton beam radiotherapy (PBT) offers great promise for the treatment of a wide variety of cancers because of the sharp decrease in radiation dose at a defined point, known as the Bragg peak [38]. PBT is an advanced form of radiotherapy, with radiation treatment delivered by accelerated proton beams rather than X-rays. A proton beam delivers some radiation to healthy tissue in reaching the tumor but very little radiation beyond the edge of the tumor being treated. This means that PBT is able to treat cancers just as effectively but delivers less radiation to other healthy parts of the body that surround the tumor. In order to have an effect on cancer cells, the photons are accelerated.

Like all charged particles, protons undergo a very rapid energy loss in the last few millimeters of penetration. These charged particles damage the DNA of cells, ultimately killing them. Nuclear reactions are mainly focal to clinical proton therapy and proton therapy research [39]. Protons may interact with the atomic nucleus via nuclear reactions inducing changes [40]. Protons can produce perturbations in gene expression, alterations in signaling and function in the cell cycle, invasion, and angiogenesis, and, in cancer, they can inhibit the progression processes of invasion and migration [41]. The physical characteristics of proton therapy have advantages over photon treatment, and the clinical procedure of proton therapy for cancer has growing significantly due to the dosimetric benefits of protons [42]. Protons are delivered with two different radiation techniques: passive scattering proton therapy (PSPT) or the pencil beam scanning technique (PBS). In PSPT, the whole tumor is irradiated, using collimators and compensators for adjustability dose. With PBS, the target volume is scanned spot-by-spot with a narrow proton beam [43].

## 8. Protective Properties of Molecular Hydrogen: Potential Antitumor Agent

As described above, as a modulator of apoptosis, H_2_ demonstrates anticancer activity and therapeutic effects in hematological diseases. On the contrary, following cancer radiotherapy, especially in the heart, ionizing radiation induces injury to normal tissues, [44]. These radiotoxic effects are mainly in relation to the production of ROS, which damages various cells. Since H_2_ is able to lessen ROS production, H_2_ therapy may be an effective treatment for acute radiation syndrome [45]. Additionally, H_2_ is a protective agent in the radioprotection of radiation-sensitive tissues, for instance, in skin cancers [46]. The use of a hyperbaric process has also shown beneficial effects in various cancer interventions. Recent data support the validity of the use of hyperbaric oxygen and hyperbaric H_2_ in the treatment of malignancies [47].

In the clinical context, there is a need for chemotherapeutic drugs without severe side-effects. One of these chemotherapeutic agents is cisplatin, which is used to treat a variety of tumors. However, high-dose therapy is limited by nephrotoxic effects [48]. Cisplatin stimulates the generation of ROS and renal lipid peroxidation. The cisplatin-associated toxicities result in part in ROS production, triggering organ injury. There has therefore been an effort to identify the protective effects of agents with antioxidant properties on cisplatin-induced adverse reactions, especially nephrotoxicity [49]. H_2_ has shown a possible protective effect in experimental studies: the inhalation of H_2_ gas and drinking H_2_ water improved mortality and weight loss caused by treatment with cisplatin and limited nephrotoxicity in mice without impairing the antitumor activity [50].

More recently, radiation treatment of liver tumors have demonstrated that drinking hydrogen-rich water may have beneficial effects on quality of life parameters. H_2_ water prevented loss of appetite, without modify the tumor response to radiotherapy [51].

On the subject of organ protection induced by H_2_, one key study investigated the possible process involved in the preventive effect of hydrogen-rich saline on doxorubicin (DOX) toxicity. The use of DOX is limited due to its cytotoxicity in the organs, such as the heart and liver [52]. In a doxorubicin rat model, it was demonstrated that a hydrogen-rich saline treatment is able to inhibit the inflammatory TNF-α/IL-6 pathway and attenuate cell apoptosis in organs such as the heart and liver [53].

Concerning malignant tumors, studies suggest these tumors can be seen as abnormal tissue containing cancer stem cells (CSCs). CSCs are a subgroup of cells within tumors. They can self-renew and produce progenitor cells of more differentiated cancer cells and are implicated in the proceeding of various tumor properties with potential new targeted therapies [54]. In mice, H_2_-rich water appears to prevent the evolution of hepatocarcinogenesis induced in mice. It was suggested that these positive outcomes were partly due to a reduction in CSCs [55]

Recent experimental results indicate that H_2_ is an antitumor agent in the treatment of glioblastoma. The invasive properties of glioblastoma are a key problem for curative treatment if a surgical resection is impracticable. The experimental in vivo studies showed that H_2_ inhalation could suppress glioblastoma tumor growth, prolonging the survival of mice with glioblastoma [56]. In a recent study, data show that, in a patient diagnosed with lung cancer with metastases, H_2_ gas monotherapy was associated with a significant effective control of tumors [57]. The H^+^-related therapy for the treatment of malignant gliomas is a new therapeutic strategy. In this context, inhibiting sodium/hydrogen exchanger 1 (NHE1) in gliomas acidifies tumor cells, while normal brain cells are not implicated, providing a novel perspective for the treatment of these malignant brain tumors [58].

In skin aging and skin cancer, current evidence suggest that some agents can provide protection against UV radiation-induced skin damage through antioxidant activity [59]. The application of H_2_-enhanced water prevented UV-induced ROS production and inhibited proinflammatory cytokine mRNAs for interleukin-6 and interleukin-1β [60]. In these conditions of H_2_-enriched water treatment, type-1 collagen synthesis was increased in samples compared to controls [61]. A recent study reported that drinking H_2-_enriched water reduced the volume and weight of endometrial tumors in a xenograft mouse model, H_2_ inducing pyroptosis, an inflammatory programmed cell death process, via ROS/NLRP3/caspase-1 pathways. This study showed that endometrial cells stimulated with H_2_ can result in cellular NLRP3 activation [62]. However, the role of ROS in clinical situations is not clear, having both tumor-promoting and tumor-suppressing functions [63].

From the perspectives of tumor promotion and suppression, studies have indicated that there is crosstalk between the regulatory mechanisms of H_2_ on cancer, apoptosis, and autophagy. In cancer biology, autophagy plays a dual role in tumor promotion and suppression and contributes to cancer-cell development and proliferation [64]. H_2_ plays a protective role by modulating autophagy in multiple diseases including cancer. H_2_ attenuates lung injury by inhibiting autophagy in the alveolar epithelial cells of septic rats through the inhibition of the p38 MAPK signaling pathway [65]. However, some of these recent studies in injury models remain controversial, and further research is required.

## 9. Conclusions

In conclusion, H_2_ has shown preventive and therapeutic effects. Pre-treatment with H_2_ is more effective than post-treatment [66]. H_2_ reduces oxidative stress, exerts anti-inflammatory effects, and acts as a modulator of apoptosis [67]. Adding to the direct effect of •OH scavenging, the biological effects of H_2_ are attributed to the modulation of signal transduction and alterations in gene expression. Many of the cellular mechanisms in H_2_ therapy remain unclear, certainly because this gas reacts with putative molecules. Furthermore, the relationship between mitochondrial energy metabolism and the distribution of H_2_ is not yet completely established [68]. The greatest advantages of using H_2_ are its ability to penetrate biological membranes and the minor adverse effects associated with its use. In these conditions, H_2,_ and different types of H_2_ donors [69] may represent novel therapeutic strategies in cancer treatment.
